# The Association Between Dental Implant and Bone Graft Failure and the Levels of Vitamin D and LDL Cholesterol: Retrospective Clinical Study

**DOI:** 10.3390/dj14080504

**Published:** 2026-08-10

**Authors:** Sara Albasarah, Bader Albader, Mishali Alsharief, Khalid Almas, Faisal E. Aljofi

**Affiliations:** 1 Department of Preventive Dental Sciences, College of Dentistry, Imam Abdulrahman Bin Faisal University, P.O. Box 1982, Dammam 31441, Saudi Arabia; 2230600021@iau.edu.sa (S.A.); msalsharief@iau.edu.sa (M.A.); kalmas@iau.edu.sa (K.A.); fealjofi@iau.edu.sa (F.E.A.); 2 University Dental Hospital, College of Dentistry, Imam Abdulrahman Bin Faisal University, P.O. Box 1982, Dammam 31441, Saudi Arabia

**Keywords:** dental implants, osseointegration, guided bone regeneration, vitamin D, low-density lipoprotein cholesterol, implant failure

## Abstract

**Background/Objectives:** Dental implant and guided bone regeneration (GBR) procedures rely on successful bone healing and remodeling. Systemic metabolic factors, including vitamin D deficiency and elevated low-density lipoprotein cholesterol (LDL-C), may adversely affect osseointegration and bone regeneration. This study aimed to investigate the association between implant/GBR failure and serum vitamin D and LDL-C levels. **Methods:** A retrospective clinical study was conducted on 40 patients treated with dental implants and/or GBR procedures between 2023 and 2025 in the Eastern Province of Saudi Arabia. Twenty patients with failed outcomes and twenty patients with successful outcomes were included. Smokers, immunocompromised patients, individuals with a history of radiotherapy or chemotherapy, and patients with uncontrolled diabetes mellitus (HbA1c > 6.5%) were excluded. Failure was defined as progressive bone loss, loss of osseointegration, implant mobility, graft resorption, or persistent infection and was categorized as early or late failure. Serum vitamin D and LDL-C levels were obtained after treatment and analyzed using bivariate and exploratory multinomial logistic regression analyses. **Results:** Significant differences in serum vitamin D and LDL-C levels were observed among failure groups. Mean vitamin D levels were highest in the non-failure group (91.3 ± 47.9 nmol/L), followed by early failure (53.3 ± 33.7 nmol/L) and late failure (28.3 ± 11.3 nmol/L) (*p* = 0.001). Mean LDL-C levels increased progressively from the non-failure group (107.0 ± 26.8 mg/dL) to early failure (137.6 ± 47.3 mg/dL) and late failure groups (161.3 ± 57.0 mg/dL) (*p* = 0.003). Vitamin D status and LDL-C category were significantly associated with failure type (*p* = 0.001 and *p* = 0.012, respectively). **Conclusions:** Lower serum vitamin D levels and higher LDL-C levels were associated with implant and GBR failure, particularly in late failure cases. However, because serum vitamin D and LDL-C levels were measured after treatment outcomes and the study was limited by its retrospective design and relatively small sample size, these findings should be interpreted with caution and do not establish a causal relationship. Larger prospective studies are needed to determine whether preoperative assessment and optimization of vitamin D and lipid status may improve implant and regenerative treatment outcomes.

## 1. Introduction

Dental implant therapy has been widely used as the optimal treatment option for partially and completely edentulous patients in replacing missing teeth. Albrektsson in 1981 reported the interrelationship of various components that ensure successful outcomes in dental implants, including the biocompatibility of the implant material, the nature of implant surface, the status of implant bed, the surgical technique, the undisturbed healing phase, and the prosthetic design and loading phase [1].

Dental implant is considered successful when it shows no mobility, no peri-implant radiolucency, annual vertical bone loss not more than 0.2 mm following the 1st year of service, and absence of persistent signs and symptoms such as pain, infection, neuropathies, and paresthesia [2]. Although dental implant success rate has been reported as 97% at 10-years follow up [3]. However, implant failure is still not avoidable, and studies have reported possible risk factors associated with early and late implant loss [4,5].

Risk factors can be divided into iatrogenic, material-associated, and patient-related factors [6].

Most of the risk factors reported include age, gender, location of the implant, bone quantity and quality, implant characteristics, and the patient’s systemic condition, mainly diabetes and smoking status [4,5,6]. Recognition of systemic risk factors is important to reduce the failure rate. Some systemic anomalies are present without the patient’s awareness. Consequently, increasing attention has been directed toward identifying potentially modifiable systemic factors that may influence bone healing and osseointegration. Early recognition and management of these factors before implant placement may improve treatment predictability and reduce the risk of implant or bone graft failure [6,7].

One of the common biological anomalies that is related to bone formation and remodeling is vitamin D deficiency. Vitamin D stimulates the activity of osteoclasts and increases the production of extracellular matrix proteins by osteoblasts [7]. It also stimulates intestinal calcium absorption and inhibits the synthesis and secretion of parathyroid hormone [7]. Vitamin D also has extensive immunomodulatory effects and a direct connection to infection risk [7]. Because of these biological functions, vitamin D has attracted considerable interest as a potential determinant of implant osseointegration and regenerative healing. Clinical studies investigating this association, however, have reported inconsistent findings. While some studies demonstrated improved implant outcomes in patients with adequate vitamin D levels, others failed to identify a statistically significant relationship, despite observing similar trends. Differences in study design, sample size, patient selection, and the timing of vitamin D assessment may partly explain these conflicting results [8,9,10].

Another common biological risk factor is the high level of Low-Density Lipoprotein Cholesterol (LDL). The bone releases enzymes that are involved in the oxidation of LDL. Therefore, it is possible that the oxidized LDL accumulated in the bone could induce subsequent deleterious cellular effects on bone density [7]. High LDL was found to cause imbalance in the bone-remodeling process, a reduction in bone mass by increasing the activity, and a differentiation of osteoclasts, and inhibition of osteoblastic activity [11,12]. In addition to its effects on bone metabolism, elevated LDL has been associated with chronic low-grade inflammation, endothelial dysfunction, and impaired tissue repair, all of which may adversely affect bone regeneration and implant healing. Despite these plausible biological mechanisms, relatively few clinical studies have evaluated the relationship between dyslipidemia and implant or guided bone regeneration (GBR) outcomes, and the available evidence remains limited [7,13,14,15]. Beyond its direct effects on bone cells, oxidized LDL has been shown to promote a persistent pro-inflammatory microenvironment by increasing the production of inflammatory cytokines and reactive oxygen species while impairing endothelial function and angiogenesis [13,14]. These changes may compromise the vascular supply and cellular events required for successful osseointegration and bone regeneration [13,14]. Experimental studies have demonstrated that hyperlipidemia is associated with reduced bone formation, impaired graft maturation, and diminished implant stability, suggesting that dyslipidemia may adversely affect the biological processes essential for implant success. Nevertheless, clinical evidence evaluating the influence of LDL cholesterol on implant and guided bone regeneration (GBR) outcomes remains scarce, and the available studies have reported inconsistent findings, highlighting the need for further clinical investigation [13,14,15].

As both vitamin D and LDL cholesterol influence bone remodeling, inflammation, and the biological processes involved in bone regeneration and osseointegration, evaluating their association with implant and GBR outcomes may improve understanding of potentially modifiable systemic risk factors. Identification of such factors could help clinicians optimize patients before implant placement and regenerative procedures, thereby potentially improving treatment outcomes. Furthermore, although previous studies have investigated vitamin D or lipid profile individually, very few clinical studies have simultaneously evaluated both biomarkers in relation to implant and GBR failure. Furthermore, both implant osseointegration and GBR rely on similar biological processes, including inflammation, angiogenesis, osteogenesis, and bone remodeling. Therefore, investigating systemic metabolic factors that may influence these shared healing mechanisms could improve understanding of patient-related determinants of treatment success. Despite growing interest in vitamin D and lipid metabolism, clinical evidence evaluating both biomarkers simultaneously in relation to implant and GBR outcomes remains scarce [15,16,17,18,19]. Therefore, the aim of this study was to investigate the association between implant and bone graft failure and serum vitamin D and low-density lipoprotein cholesterol levels. The null hypothesis was that serum vitamin D and LDL cholesterol levels would not be associated with implant or GBR failure.

## 2. Materials and Methods

Periodontists, implantologists, and Periodontic Residents from the University Dental Hospital and affiliated dental healthcare facilities in the Eastern Province of Saudi Arabia were invited via institutional email and WhatsApp messages to identify potentially eligible patients treated between 2023 and 2025. Clinical records were screened according to predefined inclusion and exclusion criteria. Patients fulfilling the study definition of implant and/or guided bone regeneration (GBR) failure were enrolled as cases. Eligible patients were subsequently contacted, recalled to the clinic, informed about the study, and written informed consent was obtained prior to participation and collection of laboratory investigations. The study was designed as a retrospective observational clinical study comparing patients with failed implant/GBR outcomes and with those demonstrating successful treatment outcomes. This study was approved by the Institutional Review Board of Imam Abdulrahman Bin Faisal University. Patients aged 18 years or older with history of failed or non-failed implant/GBR performed between 2023 and 2025 were included.

Controls were selected from the same treatment period and clinical settings and consisted of patients with successful implant/GBR outcomes who demonstrated uneventful healing and remained free of progressive bone loss, implant mobility, graft resorption, or persistent infection throughout their entire documented follow-up period. Potentially eligible patients were excluded if they had incomplete clinical records, missing laboratory investigations, met any of the predefined exclusion criteria, or declined participation after being contacted. Failure was defined as the presence of one or more of the following clinical outcomes: progressive bone loss (>50%), loss of osseointegration or implant mobility, graft resorption, or persistent infection. Failure cases were further categorized as early (within 6 months after surgery) or late (more than 6 months after surgery) failure according to the time elapsed between the surgical procedure and the occurrence of failure [5]. Follow-up records for all participants were reviewed until the most recent documented clinical visit. Follow-up duration ranged from 6 to 24 months. Patients were classified as controls only if no clinical or radiographic evidence of implant or GBR failure was identified throughout their complete documented follow-up period.

To minimize the influence of established risk factors for implant failure and reduce potential confounding, smokers, patients with uncontrolled diabetes mellitus (HbA1c > 6.5%), immunocompromised patients, and those with a history of radiotherapy or chemotherapy were excluded from the study. These exclusion criteria were selected because they represent well-established systemic factors known to adversely affect wound healing, bone metabolism, and implant osseointegration. A total of 40 patients were included in this study.

Patients’ demographic data and procedure records were collected from the medical records including patient age, gender, time of surgery, type of surgery (immediate implant, delayed implant, or guided bone regeneration), and site of surgery (maxilla or mandible). Cases with incomplete demographic, clinical, or laboratory data were excluded from the analysis. Failure incidence includes progressive bone loss (>50%), loss of osseointegration/implant mobility, graft resorption, and persistent infection. Type of failure was categorized based on the duration between the surgery and the failure (Early or Late) or No failure. The Control group consisted of the non-failed implant/GBR cases.

Patients were contacted after treatment outcomes to obtain the following laboratory tests: Vitamin D + LDL. HbA1c was obtained for all participants to verify the absence of uncontrolled diabetes mellitus (HbA1c > 6.5%), one of the predefined exclusion criteria [20]. Participants were allowed to undergo laboratory testing at any accredited clinical laboratory of their choice. Because laboratory investigations were performed at different accredited laboratories, vitamin D and LDL values were standardized to common measurement units before statistical analysis to ensure consistency across all participants. The study focused on routinely reported clinical laboratory parameters measured using standardized methodologies in accredited laboratories. Serum vitamin D concentrations reported in ng/mL were converted to nmol/L using a conversion factor of 2.5, whereas LDL cholesterol values were standardized and reported in mg/dL. Because vitamin D and LDL measurements were obtained after treatment outcomes had occurred, these laboratory values were considered indicators of the patients’ metabolic status at the time of assessment rather than at the time of implant placement or GBR surgery. Vitamin D level was categorized into optimum if (≥75 nmol/L) and non-optimum (<75 nmol/L) [21]. LDL was categorized into High (≥160 mg/dL) and Low (<160 mg/dL) [22].

Data were entered into a Microsoft Excel^®^ spreadsheet Version 16.111.2 (Microsoft Corp., Redmond, WA, USA) before statistical analysis. Continuous variables were summarized using means and standard deviations, whereas categorical variables were presented as frequencies and percentages. Bivariate analyses were performed using the Kruskal–Wallis test for continuous variables and categorical variables were compared using Pearson’s Chi-square test. When expected cell frequencies were small, the Fisher–Freeman–Halton exact test was used. Multinomial logistic regression analysis was subsequently performed to evaluate factors independently associated with early and late failure, using the non-failure group as the reference category. Variables included in the multinomial logistic regression model were selected based on their clinical relevance and previous evidence identifying them as potential risk factors for implant failure. Odds ratios (ORs) with corresponding 95% confidence intervals (CIs) were calculated. Statistical analyses were performed using IBM SPSS Statistics Version 29.0 (IBM Corp., Armonk, NY, USA), and statistical significance was established at *p* < 0.05.

## 3. Results

A total of 40 patients aged 22 to 79 years were included in the study. 20 patients (50%) demonstrated successful treatment outcomes and served as the control group. 20 patients (50%) experienced implant failure (57%) or GBR failure (43%). The most frequently recorded failure event was lack of osseointegration (30%), followed by graft resorption (25%), wound dehiscence (20%), progressive crestal bone loss (15%), and persistent infection (10%). The documented follow-up period for the study population ranged from 6 to 24 months with mean follow-up of 14.8 ± 5.3 months. The median follow-up period for the failure group was 11.5 months, while the control group was 19.0 months. All control patients remained free of clinical and radiographic signs of implant or GBR failure throughout their entire available follow-up period. To minimize the influence of established systemic risk factors, all included participants were non-smokers and had controlled HbA1c levels. Among the failure cases, 10 (25%) were classified as early failures and 10 (25%) as late failures.

Table 1 summarizes the baseline characteristics of the study population. Females represented a slight majority (52.5%), while males accounted for 47.5% of participants. Most procedures involved delayed implant placement with or without GBR (60%), and the maxilla was the most frequently treated site (57.5%). Non-optimum vitamin D levels were observed in 62.5% of participants, whereas elevated LDL cholesterol (≥160 mg/dL) was identified in 22.5% of the study population.

The overall mean age of the participants was 44.3 ± 14.8 years. Mean serum vitamin D concentration was 66.0 ± 46.3 nmol/L, while the mean LDL cholesterol level was 128.2 ± 46.4 mg/dL (Table 2). Bivariate analyses demonstrated significant differences in serum vitamin D and LDL levels across implant failure types (Table 3). Mean vitamin D concentrations were highest in the non-failed group (91.3) and progressively lower in early failure (53.3) and late failure (28.3) (*p* = 0.001). Similarly, mean LDL levels increased across failure types, with the highest levels observed in late failure (161.3) then in early failure (137.6) and the lowest in non-failed group (107) (*p* = 0.003).

**Figure 1:**
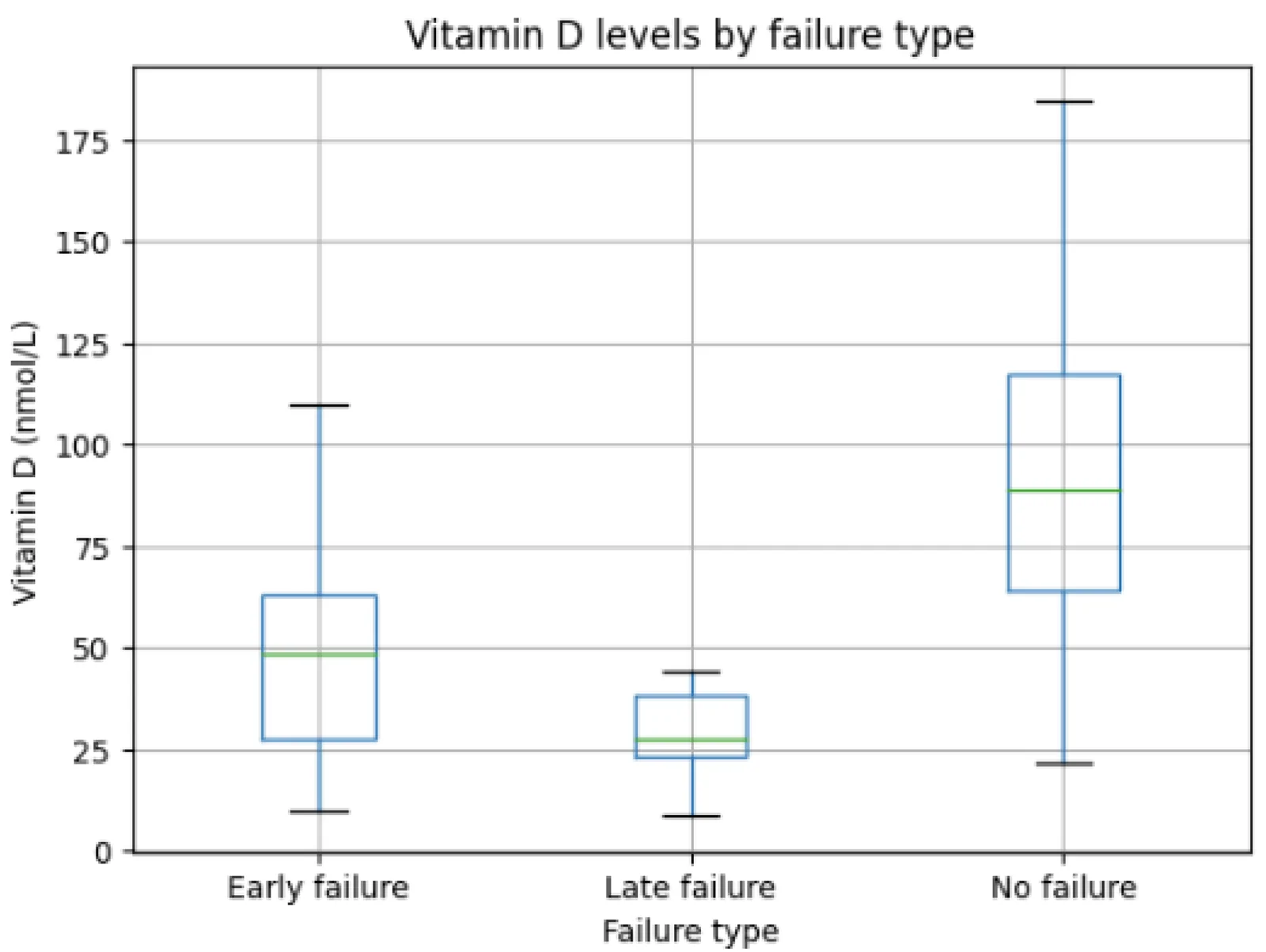
Mean (green lines) and standard deviation (blue box) of vitamin D across the study groups.

**Figure 2:**
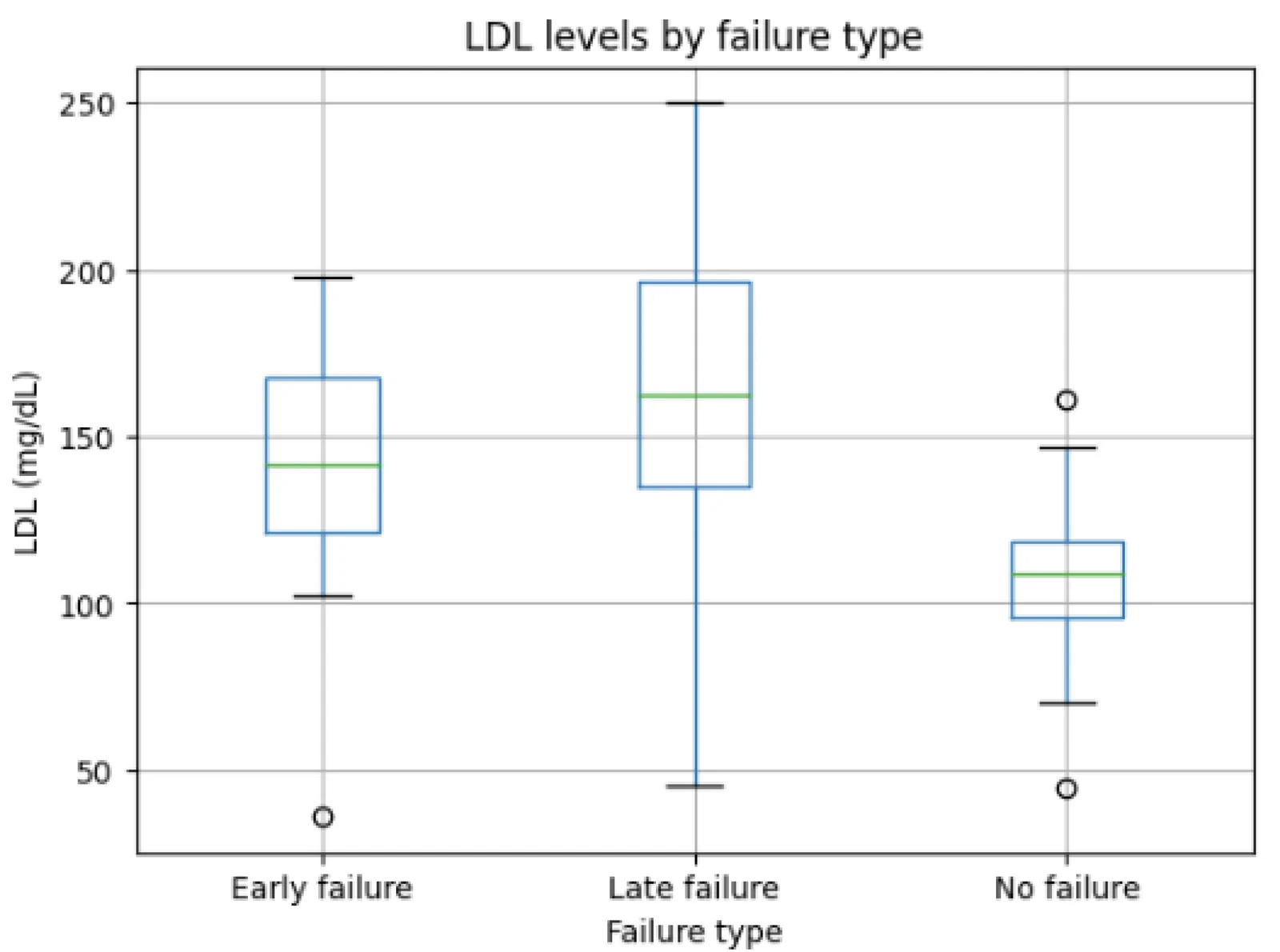
Mean and standard deviation of LDL levels across the study groups (The green lines indicate the Mean, while the blue box indicates the standard deviation, and the black lines are the range).

Bivariate analysis demonstrated significant differences in serum vitamin D and LDL cholesterol levels across failure groups (Table 3). Mean vitamin D concentrations decreased progressively from the non-failure group (91.3 ± 47.9 nmol/L) to the early failure group (53.3 ± 33.7 nmol/L) and were lowest among patients with late failure (28.3 ± 11.3 nmol/L) (Figure 1), demonstrating a significant trend toward lower vitamin D levels with increasing failure severity (*p* = 0.001). Conversely, LDL cholesterol levels increased progressively across the study groups, with the highest mean values observed in the late failure group (161.3 ± 57.0 mg/dL), followed by the early failure group (137.6 ± 47.3 mg/dL), whereas the non-failure group exhibited the lowest LDL concentrations (107.0 ± 26.8 mg/dL; *p* = 0.003) (Figure 2). No statistically significant differences were observed in participant age among the three groups (*p* = 0.260).

Categorical analysis using Pearson’s Chi-square test or the Fisher–Freeman–Halton exact test, as appropriate, demonstrated significant associations between failure type and both vitamin D status (*p* = 0.001) and LDL category (exact *p* = 0.012) (Table 4). Patients with implant/GBR failure were more likely to exhibit non-optimum vitamin D levels and elevated LDL cholesterol than patients without failure. No statistically significant associations were identified for age, gender, surgical site, or type of surgery (all *p* > 0.05). Although not statistically significant, implants placed in the maxilla showed a tendency toward a higher frequency of failure compared with mandibular implants (*p* = 0.074).

Multinomial logistic regression analysis was performed to evaluate factors associated with implant failure type, using no failure as the reference category (Table 5). Serum vitamin D status was not included in the multinomial logistic regression because the distribution of vitamin D categories across the outcome groups resulted in quasi-complete separation, preventing stable estimation of regression coefficients. Consequently, only variables that could be reliably estimated were entered into the multivariable model. Age, gender, and type of surgery were not significantly associated with either early or late implant failure (*p* > 0.05). A trend toward increased odds of late implant failure was observed for implants placed in the maxilla compared with the mandible (OR = 17.52, 95% CI: 0.85–362.18; *p* = 0.064). Similarly, patients with high LDL levels (≥160 mg/dL) showed a trend toward increased odds of late implant failure (OR = 24.12, 95% CI: 0.87–670.36; *p* = 0.061). However, neither finding reached statistical significance. No significant associations were observed between LDL category and early implant failure. Overall, demographic and surgical variables did not demonstrate statistically significant associations with failure type.

Overall, the findings demonstrated a consistent pattern whereby progressively lower vitamin D levels and progressively higher LDL cholesterol levels were associated with increasing severity of implant/GBR failure. This trend was observed across both continuous and categorical analyses and remained consistent in the regression model, although the multivariable associations did not reach statistical significance.

## 4. Discussion

The prevalence of vitamin D deficiency (<50 nmol/L) in Saudi Arabia is 81.0% [23]. The present study had 62.5% of participants with non-optimum vitamin D (<75 nmol/L). However, because serum vitamin D levels were measured after the treatment outcome had occurred, these values may not accurately represent the patients’ metabolic status at the time of implant placement or GBR surgery. Changes in vitamin D levels during the treatment period, including those related to nutritional status, supplement use, lifestyle modifications, seasonal variation, and aging, may have influenced the measured concentrations and should be considered when interpreting the findings.

The present study categorized vitamin D levels into optimum (≥75 nmol/L) and non-optimum (<75 nmol/L). The non-optimum group combined the deficiency level (<50 nmol/L) and the insufficient level (50–74 nmol/L) since previous studies reported a dose–response relationship between vitamin D and oral health where they reported better dental outcomes with higher vitamin D level not just beyond deficiency [24,25].

The prevalence of hyperlipidemia in Saudi Arabia is 54% [26]. The present study had 22% of participants diagnosed with hyperlipidemia. LDL was selected as lipid marker in this study since it is considered the most biologically relevant lipid for inflammation [16]. LDL has been reported to influence alveolar bone stability, periodontal attachment, and implant osseointegration, whereas other lipid markers have not consistently demonstrated this bone-specific effect clearly [13]. Similar to vitamin D, LDL levels were measured after treatment outcomes and therefore may not reflect the patients’ lipid status during the critical healing period following implant placement or GBR procedures.

The biological process of implant osseointegration begins with inflammation, followed by new bone formation and remodeling [16]. Similarly, GBR healing involves inflammation, osteoid formation, and bone remodeling [17]. Failure of either procedure can occur if any of these early phases are disrupted. Both procedures require immune cell recruitment, cytokine release, mesenchymal stem cell, and osteoblast differentiation. In the healing phases [1,17]. Failure may occur when inflammation is excessive or prolonged, resulting in early implant/GBR failure, or when impaired osteogenesis and bone formation compromise later stages of healing, ultimately leading to implant loss or inadequate bone regeneration [1,17,18]. Although implant failure and GBR failure represent distinct clinical outcomes, they share common biological pathways involved in bone healing and regeneration. Therefore, both procedures were analyzed together to investigate whether systemic metabolic factors, particularly vitamin D and LDL levels, may influence these shared healing mechanisms. Nevertheless, combining these procedures may have introduced clinical heterogeneity because implant osseointegration and bone graft maturation differ in their clinical endpoints and healing characteristics. This should be considered when interpreting the findings.

The present analysis demonstrated significant differences in both serum vitamin D and LDL levels across implant failure types. Non-failed implants were associated with the highest vitamin D concentrations, whereas early and late failures exhibited progressively lower levels, with the lowest value observed in late implant failure. Although these findings demonstrate an association, they should not be interpreted as evidence of a causal relationship because of the retrospective design and the timing of laboratory measurements. This graded relationship may suggest a potential role of vitamin D in modulating peri-implant bone metabolism and healing dynamics rather than acting as a binary risk factor for failure. These findings are consistent with Al-Quisi’s study where he reported statistically significant positive association between the prognosis of the dental implants regarding successful osseointegration with the vitamin D level [8]. However, their study was a prospective clinical study where they had only two cases of failed implants. Our study included 20 failed cases and investigated the vitamin D level retrospectively after the failure [8]. In contrast, another prospective study reported higher mean vitamin D levels in the early failures (106.35 nmol/L) compared with non-failed ones (79.8 nmol/L) and the difference is not statistically significant [9]. However, in their study they excluded cases with severely deficient preoperative vitamin D of <20 nmol/L, whereas in our study, the minimum vitamin D level was 8.6 nmol/L which could reflect different statistical results [9].

The present study also evaluated LDL Cholesterol and found that LDL levels differ significantly by failure type, with late implant failures exhibiting the highest lipid concentrations. Dyslipidemia has been implicated in impaired bone remodeling and altered inflammatory responses, which may contribute to compromised osseointegration or long-term implant stability. This was also reported in an animal study that reported poorer bone graft regeneration and reduced implant site stability in hyperlipidemia [14]. However, because LDL measurements were obtained after treatment outcomes, the present findings should be interpreted as associations rather than evidence that elevated LDL directly contributed to implant or GBR failure.

A recent prospective observational study on the impact of lipid profile markers and vitamin D on short-term implant survival reported strong negative correlation between vitamin D and implant loss with no statistical significance and no correlation with LDL [15]. In their study, 22.9% of the participants were smokers, and they did not mention the diabetic status [15]. This could potentially confound the results given that both are established risk factors for implant failure.

In addition, the present study found that age, gender, surgical site, and type of surgery were not significantly associated with failure type. An exploratory multinomial logistic regression analysis demonstrated trends toward increased odds of late failure among maxillary implants and patients with elevated LDL levels; these findings did not reach statistical significance and should be interpreted cautiously. Furthermore, the relatively small sample size resulted in wide confidence intervals, limiting the precision and statistical power of the multivariable analysis. Therefore, these findings should be considered hypothesis-generating rather than confirmatory, and larger prospective studies are needed to validate these observations.

This study has several limitations. First, the retrospective design precludes establishing causal relationships between serum vitamin D and LDL levels and implant/GBR failure. Although all available follow-up records were reviewed before classifying patients as controls, follow-up duration varied between 6 and 24 months with mean 14.8 ± 5.3 months. Consequently, the possibility that a small number of control patients could have developed a late complication after their final documented follow-up visit cannot be completely excluded. Second, laboratory values were obtained after treatment outcomes and may not fully reflect the patients’ biochemical status at the time of surgery. Third, the relatively small sample size limited the statistical power of multivariable analyses. An additional limitation is that laboratory investigations were performed at different accredited laboratories. Although the results were standardized to common measurement units before analysis, minor inter-laboratory analytical variation cannot be completely excluded. Finally, implant and GBR procedures were analyzed together due to similarities in biological healing mechanisms, which may introduce clinical heterogeneity. Despite these limitations, the observed associations provide preliminary evidence that systemic metabolic status may be relevant when evaluating implant and regenerative outcomes and justify further investigation in larger prospective studies.

From a clinical perspective, assessment of systemic metabolic factors such as vitamin D deficiency and dyslipidemia before implant therapy may be beneficial as part of comprehensive patient evaluation. However, based on the retrospective design of the present study, the current findings are insufficient to support routine preoperative screening or correction of these factors solely for the purpose of improving implant or GBR outcomes. Well-designed prospective studies and randomized clinical trials are required before definitive clinical recommendations can be made.

## 5. Conclusions

This retrospective clinical study demonstrated significant differences in serum vitamin D and LDL cholesterol levels between failed and non-failed dental implant and guided bone regeneration (GBR) cases. Lower vitamin D levels and higher LDL levels were associated with implant and GBR failure, particularly among late failure cases. However, because serum vitamin D and LDL levels were measured after treatment outcomes and the study was limited by its retrospective design and relatively small sample size, these findings should be interpreted with caution and do not establish a causal relationship. The present results highlight the potential relevance of systemic metabolic factors in implant and regenerative therapy and highlight the need for larger prospective studies to determine whether preoperative assessment and optimization of vitamin D and lipid status may improve clinical outcomes.

## Figures and Tables

**Table 1:** Frequency distribution of categorical variables (*n* = 40).

Variable	Category	*n*	%
Gender	Male	19	47.5
	Female	21	52.5
Vitamin D status	Non-optimum (<75 nmol/L)	25	62.5
	Optimum (≥75 nmol/L)	15	37.5
LDL category	High (≥160 mg/dL)	9	22.5
	Low (<160 mg/dL)	31	77.5
Site of surgery	Maxilla	23	57.5
	Mandible	17	42.5
Type of surgery	Immediate/Early placement	16	40.0
	Delayed placement ± GBR	24	60.0
Failure type	No failure	20	50.0
	Early failure	10	25.0
	Late failure	10	25.0

**Table 2:** Descriptive statistics of continuous variables (*n* = 40).

Variable	Mean ± SD	Minimum	Maximum
Age (years)	44.3 ± 14.8	22.0	79.0
Vitamin D (nmol/L)	66.0 ± 46.3	8.6	184.4
LDL (mg/dL)	128.2 ± 46.4	36.0	250.0

**Table 3:** Bivariate analysis of continuous variables stratified by failure type, using Kruskal–Wallis statistical test with Failure type (No failure, Early failure, Late failure) as outcome.

Variable	No Failure (Mean ± SD)	Early Failure (Mean ± SD)	Late Failure (Mean ± SD)	*p*-Value
Age (years)	41.1 ± 14.8	45.4 ± 13.5	49.7 ± 15.6	0.260
Vitamin D (nmol/L)	91.3 ± 47.9	53.3 ± 33.7	28.3 ± 11.3	0.001
LDL (mg/dL)	107.0 ± 26.8	137.6 ± 47.3	161.3 ± 57.0	0.003

**Table 4:** Bivariate analysis of categorical variables stratified by failure type. *p*-values were calculated using Pearson’s Chi-square test unless expected cell frequencies were <5, in which case the Fisher–Freeman–Halton exact test was used.

Variable	Category	No Failure *n* (%)	Early Failure *n* (%)	Late Failure *n* (%)	*p*-Value
Gender	Male	7 (35.0)	7 (70.0)	5 (50.0)	0.191
	Female	13 (65.0)	3 (30.0)	5 (50.0)	
Site of surgery	Maxilla	8 (40.0)	7 (70.0)	8 (80.0)	0.074
	Mandible	12 (60.0)	3 (30.0)	2 (20.0)	
Type of surgery	Immediate/Early	10 (50.0)	3 (30.0)	3 (30.0)	0.435
	Delayed ± GBR	10 (50.0)	7 (70.0)	7 (70.0)	
Vitamin D	Non-optimum (<75)	1 (5.0)	8 (80.0)	5 (50.0)	0.001
	Optimum (≥75)	19 (95.0)	2 (20.0)	5 (50.0)	
LDL	High (≥160)	1 (5.0)	3 (30.0)	5 (50.0)	0.012
	Low (<160)	19 (95.0)	7 (70.0)	5 (50.0)	

**Table 5:** Multinomial logistic regression analysis of factors associated with implant failure type in which type of failure is the outcome and no failure is the reference category.

Predictor	Early Failure vs. No Failure OR (95% CI)	*p*-Value	Late Failure vs. No Failure OR (95% CI)	*p*-Value
Age (years)	1.03 (0.95–1.11)	0.532	1.07 (0.96–1.19)	0.211
Gender (Male vs. Female)	2.20 (0.23–21.03)	0.492	1.28 (0.08–20.10)	0.863
Type of surgery				
Immediate/Early placement	0.46 (0.04–5.14)	0.531	0.15 (0.01–3.07)	0.218
Delayed/GBR	Reference	—	Reference	—
Site of surgery				
Maxilla	5.93 (0.60–58.39)	0.127	17.52 (0.85–362.18)	0.064
Mandible	Reference	—	Reference	—
LDL category				
High (≥160 mg/dL)	9.24 (0.47–181.24)	0.143	24.12 (0.87–670.36)	0.061
Low (<160 mg/dL)	Reference	—	Reference	—

## Data Availability

The data supporting the findings of this study are available from the corresponding author upon reasonable request.

## Abbreviations

The following abbreviations are used in this manuscript:

GBR	Guided Bone Regeneration
LDL	Low-Density Lipoprotein
HbA1c	Glycated Hemoglobin
OR	Odds Ratio
CI	Confidence Interval
SD	Standard Deviation
SPSS	Statistical Package for the Social Sciences
IAU	Imam Abdulrahman Bin Faisal University
